# Toxic Effects of Neonicotinoid Insecticide Imidacloprid and Polystyrene Microplastics on Rat Neuroblastoma B104 Cells

**DOI:** 10.3390/toxics13121060

**Published:** 2025-12-07

**Authors:** Tao Wang, Gulijiazi Yeerkenbieke, Yun Yang, Shuai Shi, Xiaoxia Lu

**Affiliations:** Ministry of Education Laboratory for Earth Surface Processes, College of Urban and Environmental Sciences, Peking University, Beijing 100871, China

**Keywords:** imidacloprid, polystyrene microplastics, combined exposure, cell proliferation, cholinergic system, oxidative stress

## Abstract

Imidacloprid (IMI) and polystyrene microplastics (PS-MPs) are common environmental pollutants, posing potential risks to ecosystems and human health. However, there is limited research on their toxic effects on nerve cells, particularly under combined exposure conditions. This study aimed to evaluate the toxic effects of IMI and PS-MPs alone and in combination on rat neuroblastoma B104 cells. Based on a cell viability assay (48 h), the No Observed Adverse Effect Levels of IMI and PS-MPs were 260 mg/L and <150 mg/L, respectively. To study their effects on the cholinergic system and oxidative stress, similar concentrations of IMI (2.6, 26, 260 mg/L) and PS-MPs (3, 30, 300 mg/L), alone and in combination, were exposed to B104 cells for 48 h. The results showed that IMI alone decreased acetylcholine (ACh) and acetylcholinesterase (AChE) contents, PS-MPs alone increased ACh and AChE contents, and under the combined condition, the effect of PS-MPs predominated over IMI. Both IMI and PS-MPs alone decreased the ratio of reduced glutathione (GSH) to oxidized glutathione (GSSG), indicating oxidative stress, and under the combined condition, the ratio of GSH/GSSG decreased more, but were less than the sum of the decreases that were observed under treatment by both compounds alone. The combined exposure exhibited antagonistic effects on all endpoints. Results of this study provides a scientific basis for the environmental risk assessment of microplastics and neonicotinoid pesticides.

## 1. Introduction

Neonicotinoid insecticides are currently the most widely used pesticides in agriculture, registered for use in over 120 countries [1]. Seven major neonicotinoids are commercially available for agricultural purposes: imidacloprid, thiamethoxam, clothianidin, acetamiprid, thiacloprid, dinotefuran, and nitenpyram. Owing to their characteristics of high efficiency, broad-spectrum efficacy, and good root absorption, they are extensively applied to protect crops, turfgrass, and trees, as well as in aquaculture [2,3]. In 2019, neonicotinoids accounted for 17.3% of global pesticide sales. China is the world’s largest producer and exporter of imidacloprid, with annual exports and production estimated at approximately 8000 tons and 14,000 tons, respectively [4]. After application, only about 5% of neonicotinoids are absorbed by the target plants, with the remainder entering environmental matrices such as soil and water. Neonicotinoid residues have been widely detected in various environmental media (soil, air, water), food items (vegetables, fruits, fish), and human biological samples (urine, blood, breast milk) [5,6,7].

Imidacloprid (IMI) belongs to the first generation of neonicotinoids and remains popular in the global pesticide market due to its high insecticidal activity. Recent studies show that IMI poses multiple potential toxic effects to mammals, particularly neurotoxicity [8,9]. For instance, IMI can alter neuronal membrane properties in mice [9]. Prenatal exposure to IMI in mice has been associated with neurobehavioral deficits in offspring and increased expression of glial fibrillary acidic protein in the motor cortex and hippocampus [8]. Kimura et al. [10] reported that IMI, at concentrations greater than 1 μM, exhibited toxicity similar to nicotine in cerebellar neurons from neonatal rats, significantly inducing excitatory Ca^2+^ influx—an indicator of neurophysiological activity shifting towards hypoactivity. Increased oxidative stress in cells may contribute to various neurological symptoms and ultimately lead to disease.

Microplastics (MPs) are plastic particles or fragments with an aerodynamic diameter of less than 5 mm. They originate from various sources such as agricultural films, soil amendments (e.g., compost, sewage sludge), and plastic products (e.g., utensils, bags). Common constituents include polyethylene, polypropylene, polystyrene, polyester, and polyamide [11]. Global plastic production reached 390.7 million tons in 2021, with Asia accounting for 52% and China contributing 32% of the total output. It is estimated that an additional 33 billion tons of plastic will accumulate on Earth by 2050 [12]. MPs are ubiquitous in the environment, having been detected in soil, water, and air. Daily intakes of MPs ranging between 0.5 and 10 μm for adults from mineral water in plastic bottles have been estimated at 40.1 μg/kg body weight (BW) per day [13]. Polystyrene (PS) is one of the most frequently detected types of microplastic [14]. Polystyrene (PS) is the fifth most widely produced plastic across the globe and has quickly expanded its market share in the production of injection-molded items, including TV enclosures, toys, cassettes, and CD jewel cases. When compared to polyethylene terephthalate (PET) and polyethylene (PE), PS has a lower recycling value and a less favorable recovery rate—factors that have led it to become one of the most prevalent polymer types in aquatic, marine, and soil ecosystems [15]. Furthermore, PS-MS of a diameter within 0.5–5 μm has been extensively employed in bioassays to investigate biological interactions and toxicity in living organisms [16,17,18].

MPs can enter organisms via ingestion, inhalation, or dermal absorption, leading to bioaccumulation and potential threats to organismal health and human safety. Nanoparticles, including MPs, can not only cause dysfunction in the digestive system after ingestion but also cross the blood–brain barrier, inducing neurotoxic effects in the central nervous system. For example, MP exposure has been shown to inhibit acetylcholinesterase activity and induce oxidative stress in the brains of European sea bass [17]. Oxidative stress has also been observed in zebrafish and mice following MP exposure [19,20]. Exposure of T98G brain cells to MPs did not induce cytolysis but significantly increased reactive oxygen species, leading to oxidative stress and neuro-cytotoxicity [21].

Neonicotinoid insecticides and MPs frequently co-exist in the environment and within organisms, where interactions between them may occur. On the one hand, MPs can adsorb neonicotinoid insecticides [22,23], and on the other hand, neonicotinoid insecticides can influence the transport of MPs [23]. Research on the combined toxic effects of co-exposure to these two pollutants is limited. Only one study has reported that combined exposure to imidacloprid and polystyrene microplastics induces greater hepatotoxicity in zebrafish than exposure to either substance alone [24].

This study aims to investigate the toxic effects of neonicotinoid insecticide IMI and polystyrene microplastics (PS-MPs) on rat neuroblastoma B104 cells under both single and combined exposure conditions. The specific objectives are as follows: (1) obtain the thresholds of the impacts of IMI and PS-MPs on B104 cells’ viability under different exposure conditions; (2) determine the impacts of IMI and PS-MPs on the cholinergic systems of B104 cells under different exposure conditions; (3) explore the impacts of IMI and PS-MPs on oxidative stress in B104 cells under different exposure conditions. The findings are expected to provide a scientific basis for environmental management and risk assessment of the emerging contaminants studied.

## 2. Materials and Methods

### 2.1. Materials and Reagents

IMI (CAS: 138261-41-3, purity > 98%) was purchased from Beijing J&K Scientific company (Beijing, China). 1 μm 50 mg/mL PS-MPs (CAS: 9003-53-6) was purchased commercially from Zhongkeleiming Technology Co., Ltd. (Beijing, China). B104 cell (RRID: CVCL 0154) was purchased from Beijing Dongfangshunke Biology Technology Company (Beijing, China). Alamar Blue detection reagent (BB-4206-1) was purchased from Beibo Biology Technology Company (Beijing, China). ACh ELISA Kit (BY-L12224A) and AChE ELISA Kit (BY-L11869A) were purchased from Baiyinglichuang Biology Technology Company (Beijing, China). A GSH and GSSG Assay Kit (S0053) was purchased from Jiuqi Biology Technology Company (Beijing, China). Dulbecco’s Modified Eagle Medium (DMEM, Cat. No. 11965092) was purchased from Qisong Biology Technology Company (Beijing, China).

### 2.2. Cell Culture Conditions and Treatments

B104 cells were cultured in 100 mm dishes using the pre-prepared complete medium, with 10 mL of the medium added to each dish. The complete medium included Dulbecco’s Modified Eagle Medium (DMEM), 10% fetal bovine serum (FBS), and 1% antibiotic–antimycotic solution. The cultures were incubated at 37 °C in a constant temperature cell incubator containing 5% CO_2_, with medium changes performed the next day. When the cells grew to occupy 80% to 90% of the surface area of the dish, cell passage was performed. During the passage, the old medium was carefully aspirated, and the cells were washed once with 5 mL of PBS buffer. Then, 1 mL of 0.25% trypsin (containing EDTA) was added to the dish and digested for 2 min until the cells detached and became rounded. New medium was then added to stop digestion, and the cells were gently pipetted to create a suspension. The cells were evenly transferred to two new dishes, and additional medium was added to achieve a total volume of 10 mL before being returned to the incubator for continued cultivation.

In the cell viability assay, the treatment concentrations of IMI were set at 130, 260, 390, 520, 650, 780, and 910 mg/L, while the treatment concentrations of PS-MPs were set at 150, 300, 450, 600, 750, 900, and 1050 mg/L. The concentrations of IMI were selected based on our previous study [25]. The concentrations of PS-MPs were set referring to the study of Tang et al. [26] with modification due to the different size of the PS and the different cells used. The concentrations for the combined exposure groups were set at 130 + 150, 260 + 300, 390 + 450, 520 + 600, 650 + 750, 780 + 900, and 910 + 1050 mg/L (IMI + PS-MPs). A control group with neither IMI nor PS-MPs was also set. Each exposure group and the control group included five replicates. The cells were incubated for 48 h.

In the assays of the cholinergic system (acetylcholine and acetylcholinesterase contents) and oxidative stress (reduced glutathione and oxidized glutathione contents), the treatment concentrations of IMI were set at 2.6, 26, and 260 mg/L, while the treatment concentrations of PS-MPs were set at 3, 30, and 300 mg/L. The concentrations for the combined exposure groups were set at 2.6 + 3, 26 + 30, and 260 + 300 mg/L (IMI + PS-MPs). The highest concentration of IMI (260 mg/L) was the No Observed Adverse Effect Level (NOAEL) determined in the cell viability assay, representing a non-lethal toxicity level. The highest concentration for PS-MPs (300 mg/L) was selected to be comparable with the IMI level. Medium and low concentrations of both pollutants were 1/10 and 1/100 of the high concentrations, respectively. The rationale for selecting these concentrations was to model “worst-case” scenarios. Human blood containing 4.935 mg/L IMI and 4.65 mg/L MPs has been reported in the literature [27,28]. A control group (blank control) with neither IMI nor PS-MPs was also set. Each exposure group and the control group included five replicates. The cells were incubated for 48 h.

For the stock solutions, IMI was dissolved in dimethyl sulfoxide (DMSO) at a concentration of 256 mg/mL, while PS-MPs were suspended in water at a concentration of 50 mg/mL. To all of the culture media, including the blank control, DMSO was added at 0.1%. The blank control contained only the complete medium and 0.1% DMSO. Table 1 shows the concentrations of IMI and PS-MPs in the culture medium under various treatments for the cholinergic system and oxidative stress assays.

### 2.3. Cell Viability Assay

Alamar Blue detection reagent was used to measure cell viability. Alamar Blue is a non-fluorescent indigo color in its oxidized state, while in its reduced state, it is reduced to a fluorescent red or pink reduction product. OD values were measured at two wavelengths: the reduced state (570 nm) and the oxidized state (600 nm). For measurement, 96-well plates were used. The growth rate per well was calculated using the following activity calculation formula:

Relative growth rate (%) = (E600 ∗ A570 − E570 ∗ A600)/(E600 ∗ C570 − E570 ∗ C600)

where

E570 represents the molar absorptivity of oxidized Alamar Blue at 570 nm = 80,586;

E600 represents the molar absorptivity of oxidized Alamar Blue at 600 nm = 117,216;

A570 represents the absorbance of the assay well at 570 nm;

A600 represents the absorbance of the assay well at 600 nm;

C570 represents the absorbance of the blank control well at 570 nm;

C600 represents the absorbance of the blank control well at 600 nm.

Inhibition rate (%) = 100(%) − growth rate (%)

### 2.4. ACh and AChE Content Assays

Intracellular ACh and AChE levels were measured using enzyme-linked immunosorbent assay (ELISA) kits (BioBYing, Beijing, China). The 96-well microplates in the corresponding kits were pre-coated with either rat acetylcholine (ACh) monoclonal antibodies or rat acetylcholinesterase (AChE) monoclonal antibodies. The detection antibodies were multifunctional, biotinylated antibodies. The sample and biotinylated antibody were added to the enzyme-labeled wells for reaction. The wells were washed with PBS buffer, and then peroxidase-labeled avidin was added for reaction. After thorough PBS washing, the substrate TMB was added for color development. TMB converted to blue under catalysis with peroxidase, and acid converted the blue to yellow. The absorbance of the sample in the reaction wells was measured using a Multiskan FC Microplate reader (Thermo Fisher Scientific, Waltham, MA, USA). The intensity of the substrate color was positively correlated with the concentration of the analyte in the sample. The final calculated result was compared with the protein concentration determined by the BCA assay for each sample to determine the relative levels of ACh and AChE in the cells of the different treatment groups.

### 2.5. Determination of Oxidative Stress Levels

Intracellular oxidized and reduced glutathione were measured using reduced glutathione (GSH) and oxidized glutathione (GSSG) detection kits (Biyotime, Shanghai, China). Glutathione reductase reduced GSSG to GSH, and GSH reacted with the chromogenic substrate DTNB (5,5′-dithiobis-2-nitrobenoic acid) to produce yellow TNB and GSSG. By appropriately preparing the reaction system and combining the two reactions, total glutathione (GSSG + GSH) became the rate-limiting factor in color production, and the amount of total glutathione determined the amount of yellow TNB formed. Therefore, the amount of total glutathione could be calculated by measuring A412 (absorbance at 412 nm). GSH in the sample was first removed using an appropriate reagent, and the GSSG content could then be determined using the above reaction principle. The GSH content was calculated by subtracting the GSSG content from the total glutathione (GSSG + GSH).

### 2.6. Statistical Analysis

All data passed the normality test, and so the Shapiro–Wilk test was used. Data are expressed as mean ± standard deviation (SD). Statistical analyses were performed using one-way ANOVA and Post Hoc Dunnett’s test (vs. control) using SPSS v.20 (IBM SPSS Inc., New York, NY, USA). A two-tailed *p* < 0.05 was considered to indicate a statistically significant difference. All reported *p*-values reflect these multiple comparison corrections, ensuring the statistical data is robust and transparently reported.

## 3. Results

### 3.1. Effects of IMI and PS-MPs on B104 Cell Viability

**Figure 1:**
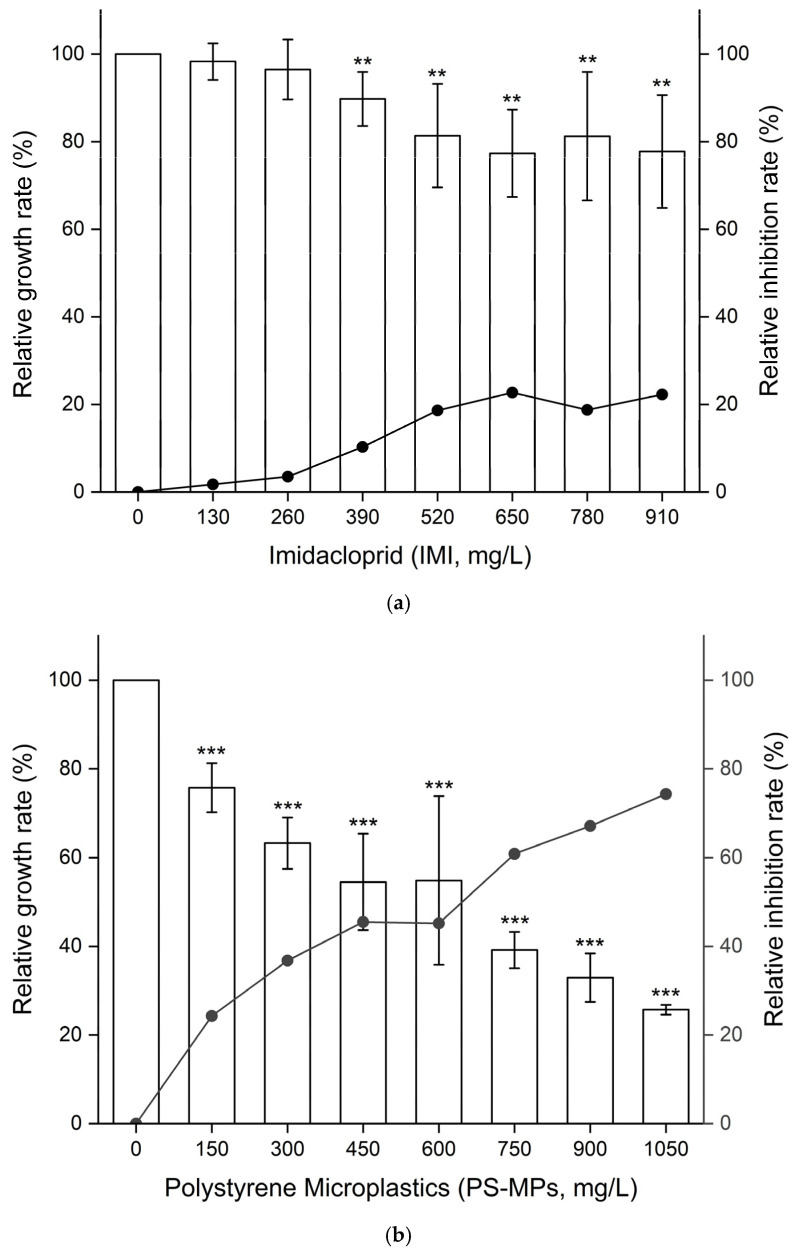

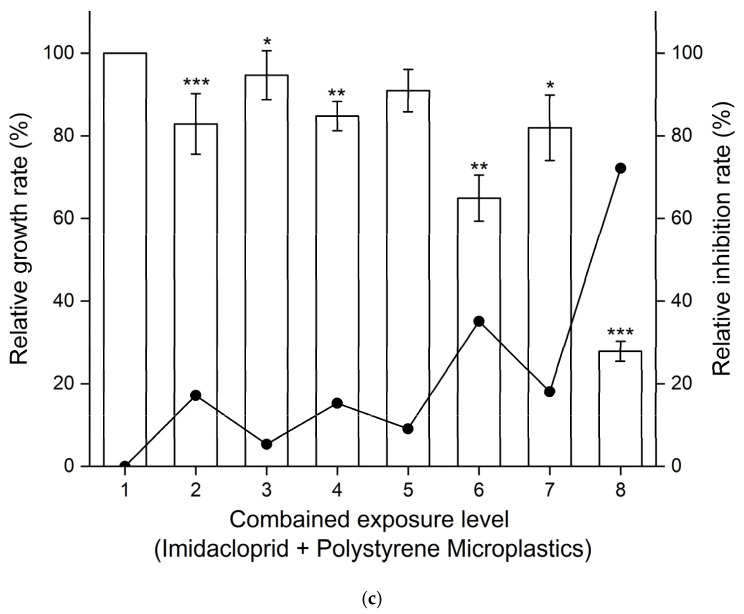
(**a**). Effects of Imidacloprid (IMI, 0–910 mg/L) on cell viability of B104 cells after 48 h of treatment. Data are presented as mean ± SD. ** indicates *p* < 0.01 (vs. control). (**b**). Effects of Polystyrene Microplastics (PS-MPs, 0–1050 mg/L) on the cell viability of B104 cells after 48 h of treatment. Data are presented as mean ± SD. *** indicates *p* < 0.001 (vs. control). (**c**). Effects of a mixture of Imidacloprid (IMI, 0–910 mg/L) and Polystyrene Microplastics (PS-MPs, 0–1050 mg/L) on the cell viability of B104 cells after 48 h of treatment. Exposure level (IMI + PS-MPs, mg/L) 1: 0 + 0, 2: 130 + 150, 3: 260 + 300, 4: 390 + 450, 5: 520 + 600, 6: 650 + 750, 7: 780 + 900, 8: 910 + 1050. Data are presented as mean ± SD. * indicates *p* < 0.05, ** indicates *p* < 0.01, and *** indicates *p* < 0.001 (vs. control).

Under the single exposure condition, after 48 h of treatment in B104 cells, the NOAEL and LOAEL values of IMI were 260 and 390 mg/L, respectively, as shown in Figure 1a. At concentrations above 390 mg/L, cell viability was significantly decreased (*p* < 0.01), and at a concentration of 910 mg/L, the relative inhibition rate of cell viability exceeded 20%. The LOAEL value of PS-MPs was 150 mg/L, with the relative inhibition rate being 24.28%, as shown in Figure 1b. At concentrations above 150 mg/L, cell viability was significantly decreased (*p* < 0.05). At a concentration above 750 mg/L, the relative inhibition rate of cell viability exceeded 50%. At a concentration of 1050 mg/L, the relative inhibition rate of cell viability reached 74%.

Under the combined exposure condition, after 48 h of treatment, the relative inhibition rate of cell viability fluctuated but had an increasing trend over the exposure levels. The overall relative inhibition rate was less than that under exposure to only PS-MPs, but higher than that under exposure to only IMI, indicating that IMI and PS-MPs had an antagonistic effect on B104 cell viability.

### 3.2. Effects of IMI and PS-MPs on ACh and AChE Contents in B104 Cells

**Figure 2:**
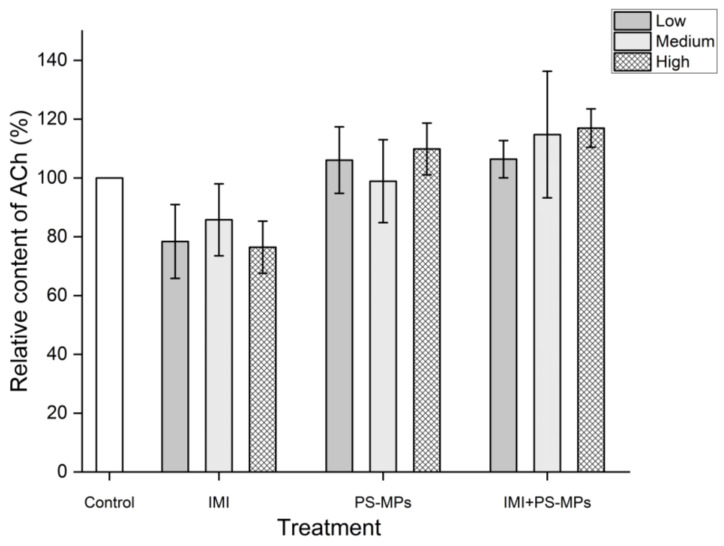
Effects of Imidacloprid (IMI, Low 2.6 mg/L, Medium 26 mg/L, High 260 mg/L) and Polystyrene Microplastics (PS-MPs, Low 3 mg/L, Medium 30 mg/L, High 300 mg/L) alone and in combination on ACh content relative to the blank control in B104 cells after 48 h of treatment. Data are presented as mean ± SD.

As shown in Figure 2, compared with the blank control, treatment with IMI alone at concentrations of 2.6, 26, and 260 mg/L decreased the relative ACh content in B104 cells by 21.60% (*p* < 0.05), 14.24%, and 23.55% (*p* < 0.05), respectively. Treatment with PS-MPs alone at concentrations of 3 and 300 mg/L increased the relative ACh content by 6.06% and 9.86%, respectively. Combined treatment with IMI and PS-MPs at the studied concentrations increased the relative ACh content in B104 cells by 6.38%, 14.77%, and 16.95%, respectively. Statistically, these increases were not significant at the 0.05 level.

**Figure 3:**
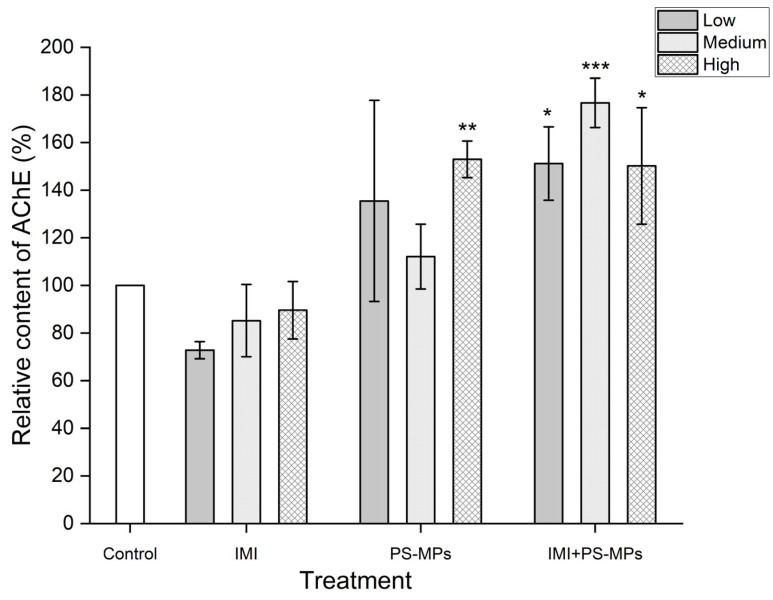
Effects of Imidacloprid (IMI, Low 2.6 mg/L, Medium 26 mg/L, High 260 mg/L) and Polystyrene Microplastics (PS-MPs, Low 3 mg/L, Medium 30 mg/L, High 300 mg/L) alone and in combination on AChE content relative to the blank control in B104 cells after 48 h of treatment. Data are presented as mean ± SD. * indicates *p* < 0.05, ** indicates *p* < 0.01, and *** indicates *p* < 0.001 (vs. control).

As shown in Figure 3, compared with the blank control, IMI treatment alone at 2.6, 26,260 mg/L concentrations decreased the relative AChE content in B104 cells by 27.20%, 14.82%, and 10.40%, respectively, but statistically, the decreases were not significant at the 0.05 level. PS-MP treatment alone at 3 and 300 mg/L increased the relative AChE content by 35.49% (*p* < 0.05) and 52.98% (*p* < 0.001), respectively. A combined treatment of IMI and PS-MPs at the studied concentrations increased the relative AChE content in B104 cells by more than 50% (*p* < 0.01).

### 3.3. Effects of IMI and PS-MPs on GSH and GSSG Contents in B104 Cells

When the body is stimulated by external factors, the highly reactive molecule ROS is overproduced. The rate of oxidative production exceeds the rate of clearance, disrupting the balance between the oxidative and antioxidant systems and leading to oxidative stress. GSH/GSSG is one of the major redox pairs in cells. The levels of GSH and GSSG, as well as the GSH/GSSG ratio, are often used as indicators of oxidative stress in organisms [29].

**Figure 4:**
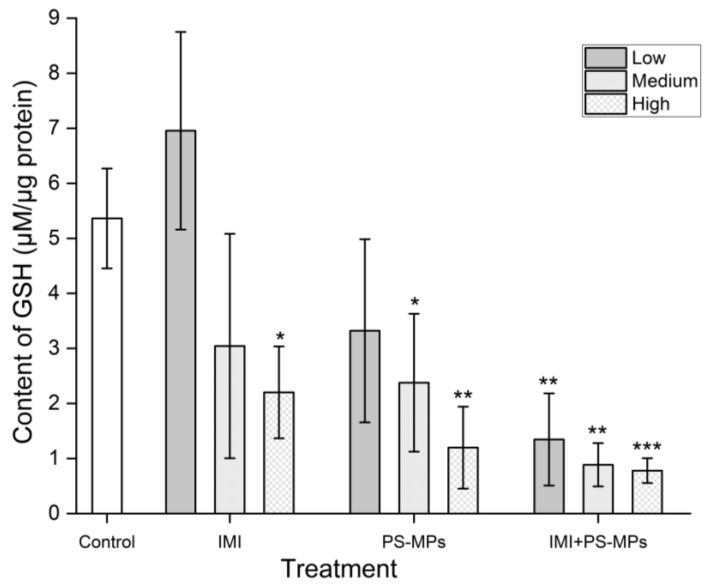
Effects of Imidacloprid (IMI, Low 2.6 mg/L, Medium 26 mg/L, High 260 mg/L) and Polystyrene Microplastics (PS-MPs, Low 3 mg/L, Medium 30 mg/L, High 300 mg/L) alone and in combination on GSH content per unit of protein in B104 cells after 48 h of treatment. Data are presented as mean ± SD. * indicates *p* < 0.05, ** indicates *p* < 0.01, and *** indicates *p* < 0.001 (vs. control).

As shown in Figure 4, intracellular GSH content was significantly decreased (*p* < 0.05) by treatment with 26 and 260 mg/L IMI (decreased by 43.24% and 58.94%), while it was increased at 2.6 mg/L IMI alone (increased by 29.70%, difference not significant at 0.05 level). Intracellular GSH content was decreased by exposure to PS-MPs alone at all the studied concentrations (decreased by 38.07%, 55.67%, and 77.66%, respectively, with all results significant at the 0.01 level). Apart from low concentrations of IMI, the decrease in GSH content was significantly positively correlated with the concentrations of IMI and PS-MPs (*p* < 0.05).

**Figure 5:**
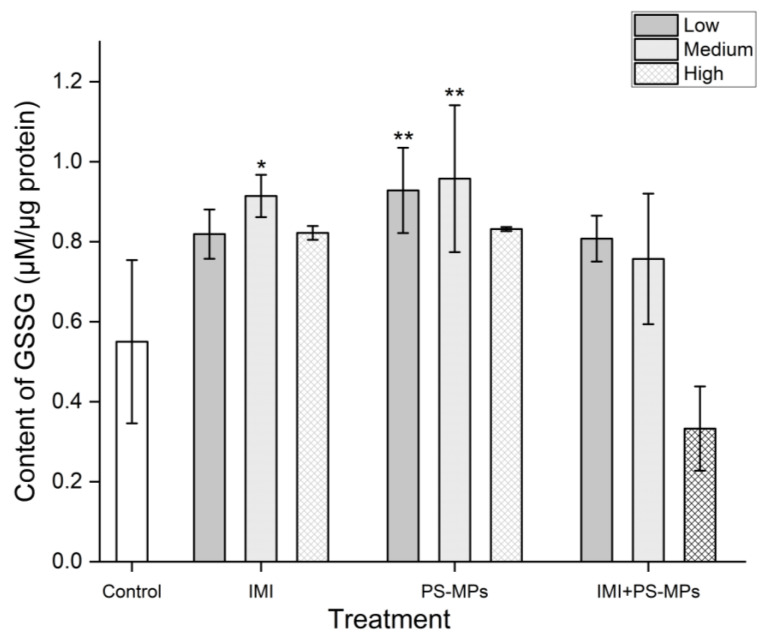
Effects of Imidacloprid (IMI, Low 2.6 mg/L, Medium 26 mg/L, High 260 mg/L) and Polystyrene Microplastics (PS-MPs, Low 3 mg/L, Medium 30 mg/L, High 300 mg/L) alone and in combination on GSSG content per unit of protein in B104 cells after 48 h of treatment. Data are presented as mean ± SD. * indicates *p* < 0.05 and ** indicates *p* < 0.01 (vs. control).

As shown in Figure 5, intracellular GSSG levels were significantly increased (*p* < 0.05) under exposure to IMI (48.90–66.23%) and PS-MPs (51.21–74.12%) alone at the studied concentrations, but no significant dose–response relationship was observed (*p* > 0.05). Under combined exposure to IMI and PS-MPs, the GSSG content was only significantly increased at the low concentration, by 46.85% (*p* < 0.05); at the medium concentration, the GSSG content was increased by 37.60% but was not significant at the 0.05 level; at the high concentration, the GSSG content was decreased by 39.48%, but was not significant at the 0.05 level.

**Figure 6:**
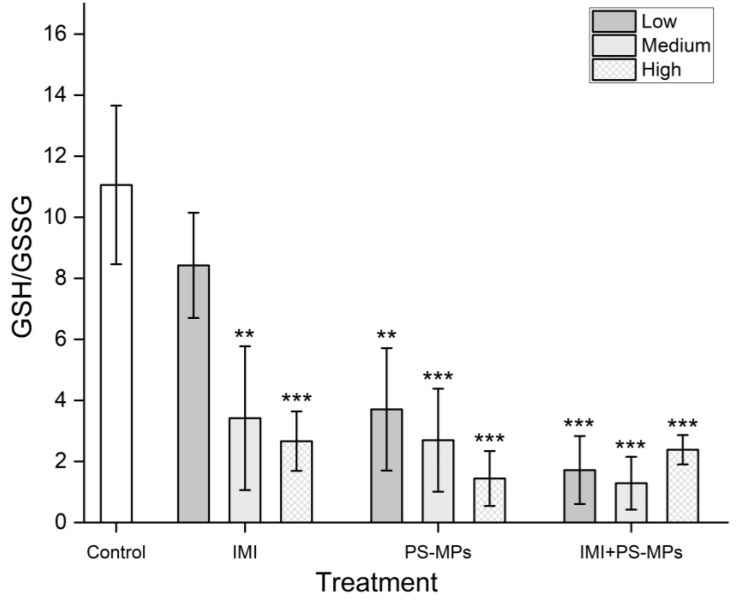
Effects of Imidacloprid (IMI, Low 2.6 mg/L, Medium 26 mg/L, High 260 mg/L) and Polystyrene Microplastics (PS-MPs, Low 3 mg/L, Medium 30 mg/L, High 300 mg/L) alone and in combination on the GSH-GSSG ratio in B104 cells after 48 h of treatment. Data are presented as mean ± SD. ** indicates *p* < 0.01, and *** indicates *p* < 0.001 (vs. control).

As shown in Figure 6, under all the exposure conditions, the intracellular GSH-GSSG ratio significantly decreased (66.49–88.35%, *p* < 0.001), apart from with low concentrations of IMI (decreased by 23.83%, but was not significant at the 0.05 level). Under exposure to IMI and PS-MPs alone, the decrease in the GSH-GSSG ratio was significantly positively correlated with the exposure concentration (*p* < 0.05). Under combined exposure to IMI and PS-MPs, no significant dose–response relationship was observed (*p* > 0.05).

## 4. Discussion

### 4.1. Impacts of IMI and PS-MPs on B104 Cells Viability

Under single exposure conditions, both IMI and PS-MPs reduced the proliferation rate of B104 cells, with a significant dose–response relationship (*p* < 0.05). IMI molecules can bind to nicotinic acetylcholine receptors (nAChRs), blocking acetylcholine (ACh) binding while preventing the termination of the nerve signal by acetylcholinesterase. As a result, nAChRs are continuously stimulated, the cholinergic system is damaged, and cells are unable to perform normal life activities, leading to cell necrosis [30,31]. The higher the concentration of IMI, the more pronounced its inhibition of cell proliferation. PS-MPs have a large surface area and rigidity. When they come into contact with the cell surface, they may directly puncture or abrade the cell membrane, compromising its integrity and causing the leakage of cellular contents, leading to cell death [32]. In addition, cells may actively internalize microplastic particles, particularly submicron and nanoparticle-sized ones. After internalization, microplastics accumulate in lysosomes, and those that cannot be degraded may cause lysosomal membrane rupture due to physical pressure or oxidative stress, releasing hydrolytic enzymes into the cytoplasm and triggering apoptosis or necrosis [33]. The higher the concentration of PS-MPs, the more pronounced their inhibition of cells becomes.

**Figure A1:**
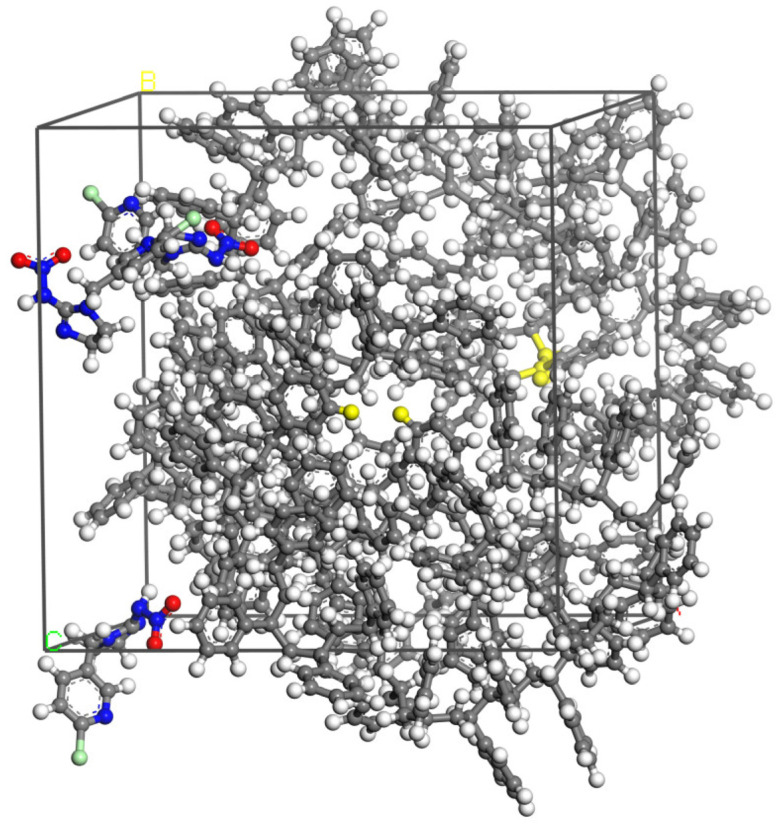
Molecular dynamics simulation of Polystyrene Microplastics (PS-MPs) and Imidacloprid (IMI). Black ball represents C, white represents H, blue represents N, red represents O, and green represents Cl.

**Figure A2:**
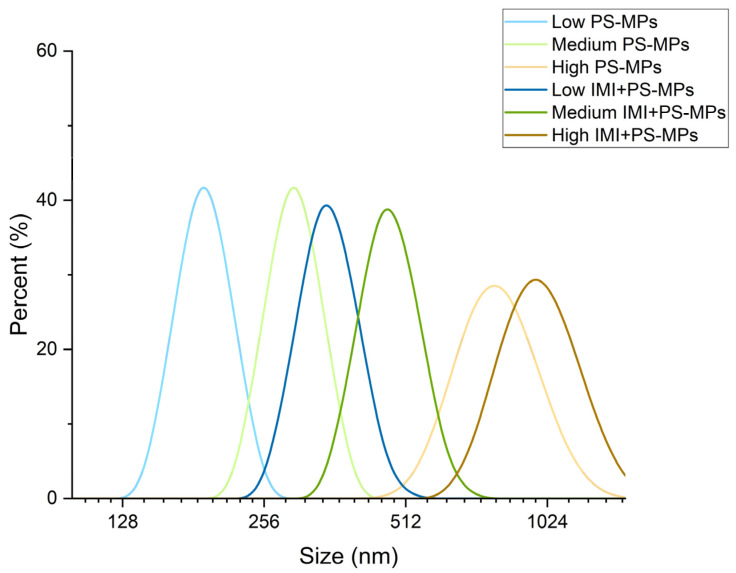
Particle size of Polystyrene Microplastics (PS-MPs) and Imidacloprid (IMI) + PS-MPs.

Under combined exposure conditions, PS-MPs, with their large surface area and hydrophobicity, can adsorb IMI molecules from the aqueous phase and concentrate them on their surfaces (Figure A1 in Appendix A.1). When cells engulf or adhere to these IMI-loaded PS-MPs, the high concentration of IMI is directly delivered to the cell interior or membrane surface, resulting in a high level of IMI in the local microenvironment or inside the cells, far exceeding that under the exposure condition of IMI alone. However, the adsorption of IMI molecules by PS-MPs also increases the particle size, significantly reducing its bioavailability. The toxicity under combined IMI and PS-MP exposure was lower than that under the single exposures, showing an antagonistic effect. This antagonism likely results from a combination of competing factors. While the adsorption of IMI onto the PS-MPs surfaces and the resulting increase in particle size (Figure A2 in Appendix A.2) can reduce the overall cellular uptake of the composite particle and lower the bioavailability of the PS-MPs, there is a competing factor. The localized delivery of a high concentration of IMI directly to the cell membrane or interior upon particle internalization also occurs. The observed antagonism, particularly at lower and medium concentrations, is therefore governed by the reduction in overall particle bioavailability, outweighing the enhanced localized IMI toxicity.

### 4.2. Impacts of IMI and PS-MPs on Cholinergic System Markers in B104 Cells

Under single exposure conditions, IMI and PS-MPs affected the cholinergic system markers ACh and AChE differently. IMI reduced the contents of ACh and AChE at all the studied concentrations, while PS-MPs increased the contents of ACh and AChE at all the studied concentrations. The reduction in ACh content by IMI is consistent with IMI acting as an nAChR agonist. IMI, by binding to nAChRs, causes sustained depolarization of the postsynaptic membrane, resulting in abnormal ACh release and depletion, thereby interfering with normal neurotransmission [30,31]. The effect of IMI exposure alone on AChE content showed a downward trend, but this reduction generally lacked statistical significance across most doses. This suggests that the B104 cells may have been engaging in compensatory regulation to maintain AChE activity. Therefore, the IMI neurotoxicity was driven by ACh depletion coupled with a tendency toward AChE suppression [34].

On the other hand, PS-MPs may affect the integrity of the cell membrane or vesicular transport through physical adsorption or membrane interference, indirectly disrupting the dynamic balance of neurotransmitters. The mild cellular stress caused by PS-MPs may trigger endogenous neuroprotective mechanisms, leading to compensatory upregulation of AChE to counter potential neurotransmitter imbalances, and may also increase ACh synthesis or release.

Under combined exposure conditions, the contents of ACh and AChE increased, similar to those observed under the condition of PS-MP exposure alone, except that the increase in AChE content under medium exposure was slightly higher and more significant. These results indicated that PS-MPs predominated in regulating AChE activity under the combined exposure condition, effectively masking the inhibitory effects of IMI. At a medium concentration, IMI adsorption on PS-MPs might have modified the surface of the PS-MPs, increasing the bioavailability of PS-MPs, and thus leading to higher toxicity. At a high concentration, PS-MPs underwent agglomeration, increasing their size and thus reducing their bioavailability and toxicity. Further research is needed to explore and confirm these mechanisms.

The PS-MPs’ mechanism of action leads to compensatory upregulation of AChE, possibly through the activation of cellular stress pathways, which is fundamentally different from IMI’s direct agonism. This active modulatory role of PS-MPs, which is triggered even by the reduced bioavailable fraction, is the primary driver of the cholinergic response in the combined exposure group, overriding the inhibitory effect of IMI.

### 4.3. Impacts of IMI and PS-MPs on Oxidative Stress Markers in B104 Cells

Under single exposure conditions, both IMI and PS-MPs significantly decreased the contents of GSH, increased the contents of GSSG, and decreased the GSH/GSSG ratio (*p* < 0.05), apart from with low concentrations of IMI. The decreases in the GSH content and the GSH/GSSG ratio by PS-MPs were higher than those by IMI, indicating higher oxidative stress induced by PS-MPs. IMI may activate nAChR receptors, causing a Ca^2+^ influx [10] and increased mitochondrial ROS. After PS-MPs are internalized by cells, they may damage lysosomal membranes, trigger oxidative bursts, increase intracellular PS-MP accumulation as a carrier for other pollutants, and activate inflammatory pathways, indirectly inducing ROS generation and antioxidant resource depletion [35,36]. Under combined exposure conditions, the GSH content and the GSH/GSSG ratio were further decreased, but the extent of this decrease was less than the sum of the decreases under both single exposure conditions, indicating that IMI and PS-MPs have antagonistic effects on oxidative stress. Under the high combined exposure condition, 260 mg/L IMI + 300 mg/L PS-MPs, the GSSG level slightly decreased, possibly due to apoptosis or necrosis occurring at extremely high concentrations, leading to a reduction in metabolic activity.

**Figure A3:**
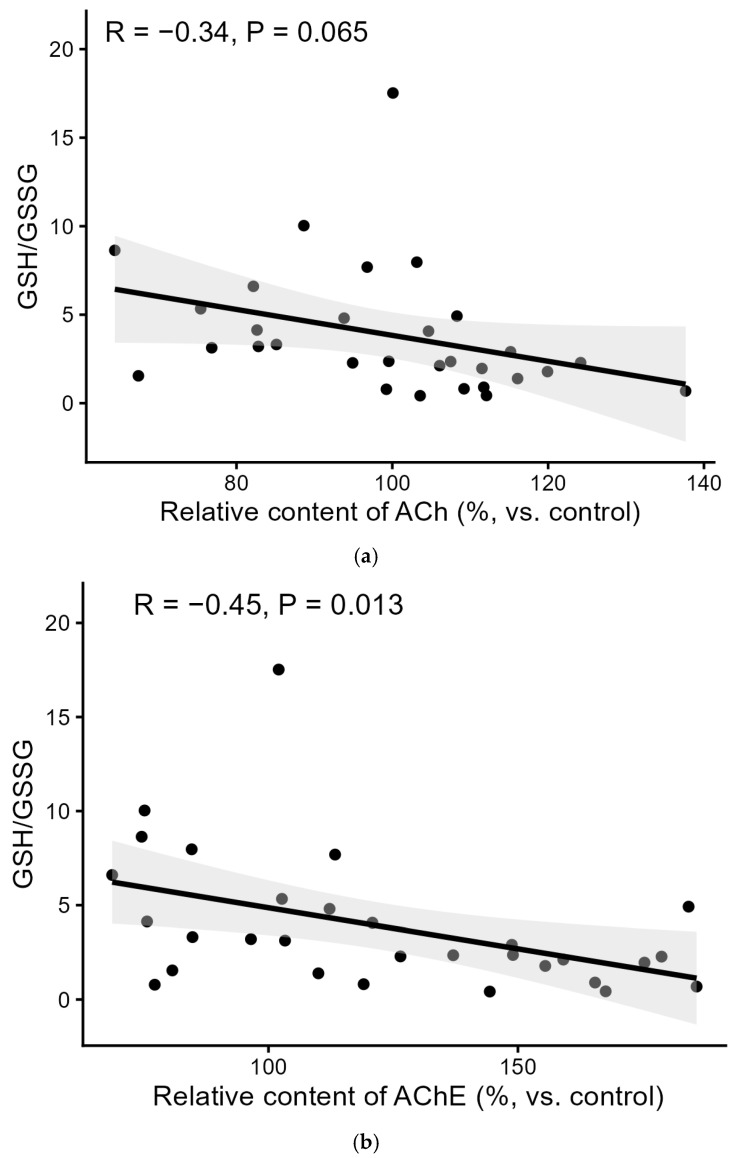
(**a**). Pearson correlation analysis between ACh and GSH/GSSG. (**b**). Pearson correlation analysis between AChE and GSH/GSSG. The gray shaded areas represent the 95% confidence intervals of the linear regressions.

A Pearson correlation analysis was performed to confirm the mechanistic role of the disruption (Figure A3 in Appendix A.3). Crucially, the GSH/GSSG ratio showed a negative correlation with ACh content (R = −0.34, *p* = 0.065) and AChE content (R = −0.45, *p* < 0.05). This compelling evidence validates the claim that oxidative stress is a primary contributor to the observed neurotoxicity, functionally linking compromised redox status to the dysregulation of the cholinergic system. This integrated finding supports a model where the antagonistic effects in combined exposure involve the mutual moderation of their impacts on this fundamental oxidative stress pathway.

### 4.4. Comparison with Available Studies

Comparisons of our findings with similar neurotoxicity studies using neuroblastoma cell lines in the literature revealed both consistencies and differences. Ramirez-Cando et al. reported that IMI (2.56–128 mg/L, exposure 7 d) significantly increased the production of reactive oxygen species (ROS) and reactive nitrogen species (RNS) in human neuroblastoma SH-SY5Y cells, leading to mitochondrial dysfunction and early apoptosis [37]. Wang et al. found that IMI (17.5 and 101.8 mg/L, exposure 24 h) caused metabolic disturbances and redox homeostasis damage in mouse neuroblastoma Neuro-2a cells [38].

Research using SH-SY5Y cells demonstrated that PS-NPs induced neurotoxicity, with oxidative stress and mitochondrial dysfunction as key mechanisms. Tang et al. reported that the NOAEL of PS-NPs (50 nm, exposure 24 h) determined by the cell viability assay was 100 mg/L, and PS-NPs under 100 mg/L and 200 mg/L linked ROS production to apoptosis and autophagy [26]. Ma et al. introduced surface-modified nanoplastics (~100 nm, 30 mg/L), showing that while all variants disrupted mitochondrial function, PS-COOH and PS-NH2 induced stronger Drp1-mediated fission and apoptosis than PS [39]. Liu et al. demonstrated the impact of particle size; at the same concentrations (5, 25, and 75 mg/L), PS-NPs (nanoplastics, 100 nm) induced more ROS production, cell cycle arrest, and reduced viability in HT22 cells than larger PS-MPs (1 μm) did [40]. Bai et al. (2024) further highlighted the impact of surface modifications, showing that PS-NH2 (100 nm) triggered apoptosis via Bax/Bcl-2 imbalance and nitric oxide synthase signaling [41]. These studies emphasized the roles of size and surface characters in PS-induced neurotoxicity.

However, the combined effects of IMI and PS-MPs on neuroblastoma cells have not been reported. Particularly, the effects of IMI and PS-MPs on the cholinergic system were little known. This is the first time that the combined effects of neonicotinoid pesticide and microplastics on neuroblastoma cells have been reported.

There were a few limitations in this study. The current study focused on establishing the primary mechanism via system-level effects (cholinergic and oxidative stress). Deeper mechanistic experiments (apoptosis/TUNEL assays, PS-MPs uptake imaging, additional cell lines) are beyond the current scope but will be the focus of future research.

## 5. Conclusions

This study systematically elucidated the effects of single and combined exposure to IMI and PS-MPs on B104 cells. Both IMI and PS-MPs alone induced significant cytotoxicity, including dose-dependent inhibition of cell proliferation, cholinergic system disruption, and oxidative stress, and their combination showed antagonistic effects. Notably, at the cholinergic neurotoxicity endpoint, IMI and PS-MPs alone had different effects; IMI decreased ACh and AChE contents, while PS-MPs increased ACh and AChE contents; under combined conditions, the effect of PS-MPs predominated over IMI. Although both IMI and PS-MPs alone inhibited cell proliferation and caused oxidative stress, the effects of their combination were less than the sum of their effects alone. This suggests that microplastics coexist with neonicotinoid pesticides; their role goes beyond being a passive carrier and becomes an active biological effect modulator. Therefore, risk assessments of coexisting pollutants in the environment must transcend the framework of individual toxicity and fully consider the complex interactions that may arise under combined exposure conditions. Future research should further explore the molecular mechanisms underlying these interactions and provide a deeper theoretical basis for accurately assessing their ecological and health risks.

## Figures and Tables

**Table 1:** Experimental setup for the cholinergic system and oxidative stress assays.

Treatment	IMI (mg/L)	PS-MPs (mg/L)
Blank control	0	0
Low IMI	2.6	0
Medium IMI	26	0
High IMI	260	0
Low PS-MPs	0	3
Medium PS-MPs	0	30
High PS-MPs	0	300
Low IMI + PS-MPs	2.6	3
Medium IMI + PS-MPs	26	30
High IMI + PS-MPs	260	300

Note: IMI refers to Imidacloprid, PS-MPs refers to Polystyrene Microplastics.

## Data Availability

The data presented in this study are available on request from the corresponding author. The data are not publicly available due to privacy.

## Abbreviations

The following abbreviations are used in this manuscript:

ACh	Acetylcholine
AChE	Acetylcholinesterase
nAChRs	Nicotinic acetylcholine receptors
GSH	Reduced glutathione
GSSG	Oxidized glutathione
IMI	Imidacloprid
PS-MPs	Polystyrene Microplastics

## Appendix A

### Appendix A.1. GCMC-MD Simulation

The model construction for PS microplastics and IMI was described as follows:

First, the PS molecular chain (Polymerization degree was 160) was constructed, and then the “Amorphous Cell” module of MS was used to construct the amorphous PS model. The IMI models were constructed based on their molecular structural formula, and the molecular configuration of the pesticide after geometry optimization was used for the subsequent adsorption calculation. Geometry optimization, annealing, and molecular dynamics simulations of the amorphous PS were carried out to get closer to the actual state, and the final configuration was used for the subsequent adsorption calculation.

All the calculations in this study were mainly completed by using the “Sorption” and “Forcite” modules of MS software (Materials Studio v. 2020). The “Sorption” module of MS software was used for the Grand Canonical Monte Carlo (GCMC) simulation. The calculation task was set to “Fixed pressure”. Sorbate was set to a geometrically optimized pesticide molecule. The adsorption temperature was set to 298 K. The Monte Carlo method was set as “Metropolis”. A total of 2 × 10^6^ steps were calculated in the equilibrium stage, and 2 × 10^6^ steps were calculated in the data output stage. The energy calculation parameters followed Table A1. The “Forcite” module of the MS software was used for MD simulation. The system was set to NVT. The initial velocity was set to a Boltzmann distribution. The simulation temperature was set to 298 K. The time step was 1 fs, and the total simulation duration was 200 ps. The result configuration was output every 2 ps, and 101 frames were output in total. A pesticide molecule was placed near the surface of the pretreated PS model, and a vacuum layer of 50 Å was added to prevent the molecules in the model from interacting with their periodic mirror images. The results of the molecular dynamics were obtained after the system reached equilibrium. The calculation followed the method reported in the literature [42,43].

**Table A1:** Energy parameters setting.

Calculation Parameters	Setting
Field	COMPASS II
Charge calculation method	Forcefield assigned
Van Der Waals Force calculation method	Atom based
Calculation of truncation radius by Van Der Waals Force (Å)	12.5
Electrostatic force calculation method	Ewald
Electrostatic force calculation accuracy (kcal/mol)	1 × 10^−3^

### Appendix A.2. Particle Size Analysis

The particle size distribution of single PS-MPs and combined IMI + PS-MPs was determined using a Laser Particle Size Analyzer (Malvern Zeta sizer Nano ZS, Malvern Panalytical, Malvern, Worcestershire, UK). PS-MPs suspensions (3, 30, and 300 mg/L) and the combined IMI + PS-MPs suspensions (Low, Medium, and High) were prepared in the cell culture medium. Prior to measurement, the samples were gently sonicated to ensure adequate dispersion and then loaded into the analyzer’s measurement cell. Measurements were conducted at 37 degrees to simulate the cell culture conditions. The mean particle size and the percentage distribution for each treatment group were recorded and analyzed.

### Appendix A.3. Pearson Correlation Analysis

The Pearson correlation coefficient (r) was the statistical test utilized to determine the strength and direction of the linear relationship between the GSH/GSSG ratio (the independent variable representing redox status) and the functional outcomes: ACh content and AChE content (the dependent variables). The analysis encompassed data from all single and combined exposure groups, with significance interpreted based on the r value and a threshold of *p* < 0.05. This methodological approach provides the statistical foundation for the claims made in the Discussion.
